# Comparative analysis of different preservation techniques for the storage of *Staphylococcus* phages aimed for the industrial development of phage-based antimicrobial products

**DOI:** 10.1371/journal.pone.0205728

**Published:** 2018-10-11

**Authors:** Eva González-Menéndez, Lucía Fernández, Diana Gutiérrez, Ana Rodríguez, Beatriz Martínez, Pilar García

**Affiliations:** Departamento de Tecnología y Biotecnología de Productos Lácteos, Instituto de Productos Lácteos de Asturias–Consejo Superior de Investigaciones Científicas (IPLA-CSIC), Villaviciosa, Asturias, Spain; Institute of Immunology and Experimental Therapy, Polish Academy of Sciences, POLAND

## Abstract

Bacteriophages have been proven as effective antimicrobial agents in the treatment of infectious diseases and in other biocontrol applications including food preservation and disinfection. The extensive use of bacteriophages requires improved methodologies for medium- and long-term storage as well as for easy shipping. To this aim, we have determined the stability of four *Staphylococcus* phages (phiIPLA88, phiIPLA35, phiIPLA-RODI and phiIPLA-C1C) with antimicrobial potential at different temperatures (20°C/25°C, 4°C, -20°C, -80°C, -196°C) and during lyophilization (freeze drying) using several stabilizing additives (disaccharides, glycerol, sorbitol and skim milk). Differences between phages were observed at different temperatures (20°C/25°C, 4°C and -20°C), where phages were less stable. At lower temperatures (-80°C and -196°C), all phages showed good viability after 24 months regardless of the stabilizer. Differences between phages were also observed after lyophilization although the addition of skim milk yielded a dry powder with a stable titer after 24 months. As an alternative to facilitate storage and transportation, phage encapsulation has been also explored. Phage phiIPLA-RODI encapsulated in alginate capsules retained high viability when stored at 4°C for 6 months and at 20°C for 1 month. Moreover, the spray-dryer technique allowed obtaining dry powders containing viable encapsulated phages (phiIPLA-RODI and phiIPLA88) in both skim milk and trehalose for 12 months at 4°C. Storage of phages at 20°C was less effective; in fact, phiIPLA88 was stable for at least 12 months in trehalose but not in skim milk, while phiIPLA-RODI was stable only for 6 months in either stabilizer. These results suggest that encapsulated phages might be a suitable way for shipping phages.

## Introduction

Bacteriophages are the natural enemies of bacteria. Lytic bacteriophages infect specifically a host bacterium and multiply inside the cytoplasm to finally lyse the cell. This antimicrobial property may be used as an alternative to classical antibiotics to treat human infections provoked by multi-resistant bacteria or “super-bugs” [1]. Indeed, there is an urgent need of new antimicrobials as was clearly indicated by the United Nations General Assembly [2]. In addition, there are other areas of application of bacteriophages including animal husbandry, veterinary medicine and agriculture [3]. Moreover, bacteriophages have also been proposed as new tools to improve food safety by controlling pathogenic bacteria in food [4].

Regarding the application of bacteriophages in the treatment and prophylaxis of infectious diseases in animals and humans, there are numerous studies that confirm their effectiveness. For instance, successful results were obtained in murine models of lung [5], wound [6], and gastrointestinal infections [7]. Additionally, phages have been proposed to control foodborne pathogens by reducing colonization by zoonotic bacteria in farms, to remove biofilms from industry equipment and food-contact surfaces, and to inhibit the growth of pathogenic bacteria in foods [8].

Indeed, several phage products for human therapy are available in Eastern European countries (Pyophage and Intestiphage preparations from the Eliava Institute, Tbilisi, Georgia) and products for food-related applications (ListShield^™^, PhageGuard Listex, EcoShield^™^ and SalmoFresh^™^) are currently marketed in the USA and Western Europe.

This renewed interest in bacteriophage applications has resulted in a number of *in vitro* studies about preservation and delivery of phages (recently reviewed by [9]). Some preservation techniques such as lyophilization have been used to obtain formulations suitable for clinical application of phages. For example, aerosolizing powders containing bacteriophages KS4-M and FKZ were prepared for lung delivery and treatment of *Burkholderia cepacia* and *Pseudomonas aeruginosa* infections [10] and lyophilized inserts harboring *Staphylococcus aureus* bacteriophages were used to eradicate MRSA from the nose [11]. Moreover, oral administration of phages requires enhanced resistance to the harsh gastric conditions, which have been solved by microencapsulation of phages in polymeric matrices such as alginate and pectin [12, 13]. Microencapsulation has been recently proposed for obtaining microcapsules containing phages that, once added to a propylene glycol gel, could be used as sanitizers in the food industry [14]. This study concluded that bacteriophage susceptibility to storage and processing conditions differs among phages. Therefore, these parameters should be optimized for each phage in order to guarantee the shelf life of phage-based products and ensure application of the right dosage.

In this context, we are interested in *Staphylococcus* phages and their utilization as antimicrobials to combat *S*. *aureus* and *Staphylococcus epidermidis*. Methicillin-resistant *S*. *aureus* (MRSA) strains are responsible for serious and difficult-to-treat human infections [15]. *S*. *aureus* is also one of the major bacterial agents causing foodborne diseases in humans due to the production of enterotoxins [16]. Promising results were obtained using phages against *S*. *aureus* infections in animal models of diabetic foot infections [17], diabetic cutaneous wounds [18], septicaemia [19], and chronic osteomyelitis [20]. Several studies have also demonstrated the efficacy of bacteriophages to control *S*. *aureus* development in several foods such as cheese [21, 22] and milk [23]. Phages are also able to prevent or reduce *S*. *aureus* biofilm formation [24–26].

In spite of all these studies, only one phage-based product against *Staphylococcus*, Pyophage, has been placed on the market to date. Partly, this is due to the regulatory constraints that surround marketing of phage-based products. In order to overcome these regulatory hurdles, more research is needed to provide additional evidences regarding the efficacy, safety and shelf-life stability of these preparations. For instance, it is important to determine the optimal storage conditions to ensure long-term phage stability prior to the widespread commercialization of phage-based products.

In this study, we have evaluated the stability of different *Staphylococcus* bacteriophages under different storage conditions over a 24-month period. To do that, we used four phages previously isolated in our laboratory. Two of them, phiIPLA35 and phiIPLA88, were selected as virulent mutants of the *S*. *aureus* temperate phages ΦA72 and ΦH5, respectively [27, 28]. These bacterial viruses belong to the *Siphoviridae* family and effectively inhibit *S*. *aureus* growth in dairy products [21, 27, 29, 23]. More recently, we have isolated and characterized two *Myoviridae* phages, phiIPLA-RODI and phiIPLA-C1C, infecting a broad range of staphylococcal species [25]. Their high lytic ability against planktonic cultures and biofilms makes them good candidates for removing *Staphylococcus* in both hospital and food-related settings. Our results will be useful for developing an easy and effective methodology to preserve phage stocks that can be later applied after large-scale propagation in an industrial setting. More specifically, we focused on assessing the impact of temperature and presence of a stabilizing agent on phage stability, and explored two encapsulation techniques that would allow shipping and storage without refrigeration. Additionally, we have assessed the efficacy of preparing frozen stocks for phage storage inside infected cells. Although this method would not be suitable for phage delivery to food or phage therapy applications, it would be a convenient technique for preservation of master stocks for industrial production of phages.

## Material and methods

### Bacterial strains and bacteriophage propagation

*Staphylococcus aureus* Sa9 was used as the host strain of phages phiIPLA35 and phiIPLA88 [28]. Bacteriophages phiIPLA-RODI and phiIPLA-C1C were propagated on *S*. *aureus* IPLA1 and *S*. *epidermidis* F12, respectively [25]. All strains were routinely cultured in tryptic soy broth (TSB; Scharlau, Barcelona, Spain) at 37°C with shaking or on TSB plates containing 2% (w/v) bacteriological agar (TSA). Bacteriophages were propagated as described previously [28, 25]. Briefly, early exponential cultures of *Staphylococcus* host strains (OD_600_ = 0.1) were infected with the phage at a multiplicity of infection (MOI) of 1–10. The infected cultures were then incubated for 3 h at 37°C with vigorous shaking. Phage lysates were obtained by centrifugation of cultures and subsequent filtration (0.45 μm cellulose acetate filters). Partial purification was obtained after precipitation of phage lysates by adding NaCl (0.5 M, final concentration) and PEG 8000 (10%, final concentration). Samples were maintained for 18 h at 4°C, and then centrifuged at 10,000 rpm, 30 min at 4°C. The pellet containing the phages was resuspended in SM buffer (20 mM Tris HCl, 10 mM MgSO_4_, 10 mM Ca(NO_3_)_2_ and 0.1 M NaCl, pH 7.5).

Phage titer was calculated by the plaque assay following the double-layer technique [30]. Aliquots (0.1 ml) of *Staphylococcus* stationary cultures (~10^8^ CFU/ml) were mixed with several dilutions of individual phage suspensions in SM buffer and then added to 3 ml of molten TSB top agar (0.7% w/v). This mixture was poured onto TSA plates and incubated for 18 h at 37°C. The assays were performed in triplicate and the results were expressed as PFU/ml. In the culture conditions described above, the titer of phiIPLA35 and phiIPLA88 phage lysates ranged from 10^9^−10^10^ PFU/ml, whereas phiIPLA-RODI and phiIPLA-C1C lysates showed values of 10^8^−10^9^ PFU/ml.

### Preparation of samples for low-temperature storage

Phage lysates were mixed with stabilizers resulting in a final concentration of 0.8 M trehalose, 0.8 M sucrose, 15% glycerol or 11% skim milk, and a final titer ranging from 10^8^ to 10^9^ PFU/ml. Samples before storage at low temperature were taken as control samples. Cryopreservation vials were filled with 1 ml of the above-mentioned mixtures (phage lysate plus stabilizer) and stored at -20°C, -80°C or -196°C (liquid nitrogen).

In addition, samples containing freshly infected cells were prepared as previously described Golec et al [31], with some modifications. Exponential cultures of the host staphylococcal strains (OD_600_ = 0.1) growing in TSB medium were infected with the appropriate phage at an MOI = 5 and incubated for 15 min at 20°C without shaking. Aliquots of the infected culture were immediately plated to determine the phage titer by the double layer technique. Additionally, samples were stored at -20°C, -80°C and -196°C in the presence of glycerol (15% final concentration).

All samples were stored in triplicate. At 1, 6, 12 and 24 months aliquots were tested for phage titer as described above. Aliquots of phage lysates in TSB without stabilizers were also kept in triplicate at 4°C and 20°C/25°C and the titer measured at 1, 3 and 6 months.

### Lyophilization

Phage lysates were diluted 1:1 (v/v) in 22% skim milk, 1.6 M sucrose or 30% sorbitol and frozen at -80°C in 2 ml vials for 24 h. Freshly infected cells, prepared as indicated above, were centrifuged and suspended in skim milk (11% final concentration) or sucrose (0.8 M final concentration) and immediately frozen at -80°C. Samples were lyophilized using an Alpha 1–4 freeze-dryer (Christ, Osterode am Harz, Germany) and vials were then sealed under vacuum conditions and stored at 4°C. Phage titer was determined by using the double layer technique after reconstitution of each vial with 2 ml of sterile water.

### Encapsulation in alginate

Phages were partially purified as described above and then diluted ten-fold in 50 mM HEPES pH 7.5 containing 2% (w/v) sodium alginate (FDA 21 CFR Sigma–Aldrich, USA). Samples were stirred at 500 rpm for 1 h at 20°C. Capsules were formed by dropping the phage suspension with a pipette (3.5 ml, 1 mm diameter, SARSTEDT, Germany) into a 0.1 M CaCl_2_ solution with continuous stirring. Alginate capsules were kept in this solution for 30 min at 20°C and then washed four times with milli-Q water and stored at 4°C.

Microcapsules were prepared by emulsification. The internal phase was prepared by adding 2 ml of phage suspensions to 4 ml 50 mM HEPES pH 7.5, and further mixed with sodium alginate to reach a final concentration of 3% (w/v). This solution was stirred for 1 h at 20°C with 3 ml of 30 mM CaCl_2_. The external phase of the emulsion was prepared by mixing 20 ml of Miglyol 812 (Acofarma, Spain) containing 3% (w/v) Span 80 (Sigma–Aldrich, USA) and 50 μl of glacial acetic acid; this phase was stirred for 30 min at 50°C. In order to obtain the microemulsion, the internal and external phases were homogenized at 20,000 rpm for 2 min by using a Heidolph SilentCrusher M (Merck KGaA, Darmstadt, Germany). 50 μl of glacial acetic acid were added during the homogenization step to improve gelation. Microcapsules size was determined by Dynamic Light Scattering (DLS) using a Malvern Mastersizer S Long Bench (Malvern Instruments, UK).

Triplicate samples of encapsulated and microencapsulated phages were stored at 4°C and 20°C/25°C, and aliquots were taken at 1, 2 and 3 months. Alginate capsules and microcapsules (1 g) containing phage particles were dissolved in 9 ml of 0.1 M sodium citrate for 20 min with shaking, prior to serial dilution. Sodium citrate acts by destabilizing the calcium alginate structure through exchange of calcium ions for sodium ions [32]. Phage titer was calculated as described above.

### Spray-drying

To test the effect of high temperature on phage stability, partially purified phages were diluted in SM buffer, trehalose (15% final concentration) or skim milk (11% final concentration). Samples were incubated for 30 min at different temperatures (40–60°C) and then titrated using the double-layer technique. All assays were performed in triplicate.

Mixtures of phages and stabilizers (trehalose and skim milk) were dried using a LabPlant Spray Dryer SD-05 (Keyson Products, Essex, England). For this purpose, samples (150 ml) were loaded at 40°C a flow rate of 5 ml/min, with airflow of 54 m^3^/h or 900 l/min (the inlet and outlet air temperature was 170 °C and 120°C, respectively) and compressor air pressure was 0.9 bar. The dried powder was collected and stored at 4°C and 20°C/25°C. Samples containing trehalose were stored in a desiccator with silica gel.

Powder samples containing phages were reconstituted with sterile water to reach the original volume and concentrations of skim milk and trehalose. Serial dilutions were plated for phage titration as described above.

### Statistical analysis

Statistical analysis was performed using the SPSS-PC 23.0 software (SPSS, Chicago, IL, USA), in order to establish any significant differences in phages titers among the stabilizing additives, storage conditions and time. The differences in phage titers, expressed as the mean ± standard deviation of three biological replicates in all the assays, were determined by one-way analysis of variance (ANOVA). The Student-Newman-Keuls test was used for a comparison of means at a level of significance of P<0.01 or P<0.05 depending on the experiment.

## Results

### Low-temperature storage improves preservation of *Staphylococcus* phages

With the aim of finding the most suitable conditions for storage of phages phiIPLA35, phiIPLA88, phiIPLA-RODI and phiIPLA-C1C, samples were stored in different conditions for 24 months and the titer evaluated along this time.

Phage phiIPLA88 showed good stability at 4°C with a reduction of less than 1 log unit, while the rest of phages were less stable, with reductions of about 3, 2 and 1 log units, for phiIPLAC1C, phiIPLA-RODI and phiIPLA35, respectively (Table 1).

**Table 1:** Stability of *Staphylococcus* phages (phiIPLA35, phiIPLA88, phiIPLA-RODI and phiIPLA-C1C) stored at different temperatures in TSB without additives, and in the presence of several stabilizing additives.

Tª	Stabilizing additives	Phage titer reduction			
		phiIPLA35	phiIPLA88	phiIPLA-RODI	phiIPLA-C1C
20°C/25°C	TSB	2.61±0.27*	1.98±0.37*	4.30±0.23*	4.41±0.16*
4°C	TSB	1.10±0.05*	0.33±0.02*	2.28±0.01*	2.94±0.03*
-20°C	0.8 M Trehalose	0.09±0.01	0.10±0.09	-*	-*
	0.8 M Sucrose	0.17±0.03*	0.20±0.02*	-*	-*
	15% Glycerol	0.02±0.08	0.00±0.06	0.81±0.08*	3.60±0.00*
	11% Skim milk	0.08±0.01	0.08±0.09	2.97±0.08*	3.45±0.07*
	Infected cells	0.90±0.39*	2.35±0.58*	4.06±0.26*	3.61±0.27*
-80°C	0.8 M Trehalose	0.18±0.12	0.09±0.08	0.00±0.07	0.45±0.07*
	0.8 M Sucrose	0.07±0.01	0.07±0.07	0.02±0.13	0.47±0.08*
	15% Glycerol	0.23±0.01*	0.18±0.01	0.64±0.06*	0.29±0.10*
	11% Skim milk	0.05±0.05	0.08±0.11	0.03±0.10	0.02±0.05
	Infected cells	0.89±0.35*	0.92±0.46*	1.02±0.05*	0.68±0.08*
-196°C	0.8 M Trehalose	0.42±0.02*	0.33±0.07*	0.29±0.11*	1.31±0.10*
	0.8 M Sucrose	0.47±0.17*	0.28±0.08*	0.48±0.07*	1.06±0.05*
	15% Glycerol	0.19±0.04	0.21±0.05	0.24±0.06	1.04±0.24*
	11% Skim milk	0.22±0.01	0.34±0.10*	0.24±0.06*	1.20±0.03*
	Infected cells	0.59±0.23	0.93±0.33*	0.52±0.09*	1.23±0.31*

Note: The results, depicted as loss of phage viability, were calculated by the following expression: Mean log_10_ phage titer reduction = log_10_ [Initial phage titer (PFU/ml)/Final phage titer (PFU/ml)] ± standard deviation of three biological replicates. Storage during 24 months (4°C, -20°C and -80°C), 12 months (-196°C) and 6 months (20°C/25°C).

(*) The asterisk indicates a significantly different final phage titer compared with the initial phage titer, determined by S-N-K test (P<0.01). (-) Below the bacteriophage detection threshold (10^2^ PFU/ml).

Remarkably, samples stored at 20°C/25°C lost viability after 6 months, as titer declined up to 4.4 log units. Regarding the stability of phages at the different freezing temperatures, siphophages phiIPLA35 and phiIPLA88 showed good stability for all stabilizers, with no major changes in phage titer after 24 months at -20°C (loss of viability < 0.2 log unit). By contrast, when these phages were stored as infected cells, a loss of up to 2.4 log units was observed in the phage titer after 24 months for phage phiIPLA88 (Table 1). A lower stability was observed for myophages phiIPLA-RODI and phiIPLA-C1C at -20°C. Indeed, no lysis plaques were detected in samples stored for 24 months when trehalose or sucrose was used as stabilizers (Table 1). However, phiIPLA-RODI showed greater stability in glycerol than phiIPLA-C1C, retaining a titer of 10^8^ PFU/ml (loss of viability < 0.9 log unit) after 24 months at -20°C, while phiIPLA-C1C showed a titer of about 10^4^ PFU/ml (loss of viability ~ 3.6 unit log). Regarding the myophages stored inside host cells, samples retained a phage titer of about 10^3^–10^4^ PFU/ml (loss of viability ~ 4 unit log) after 24 months (Table 1).

The results shown in Table 1 suggest that phage titers were not substantially reduced in samples stored at -80°C after 24 months, regardless of the stabilizer used. Thus, the reductions were always below 1 log unit after 24 months for both siphophages and myophages. Similar results were observed for samples stored in liquid nitrogen after 12 months. They all maintained a high stability with the exception of phiIPLA-C1C (Table 1), for which the phage titer reduction was about 1–1.4 log units in the presence of all stabilizers.

### Lyophilization in skim milk is an alternative for phage storage

**Fig 1:**
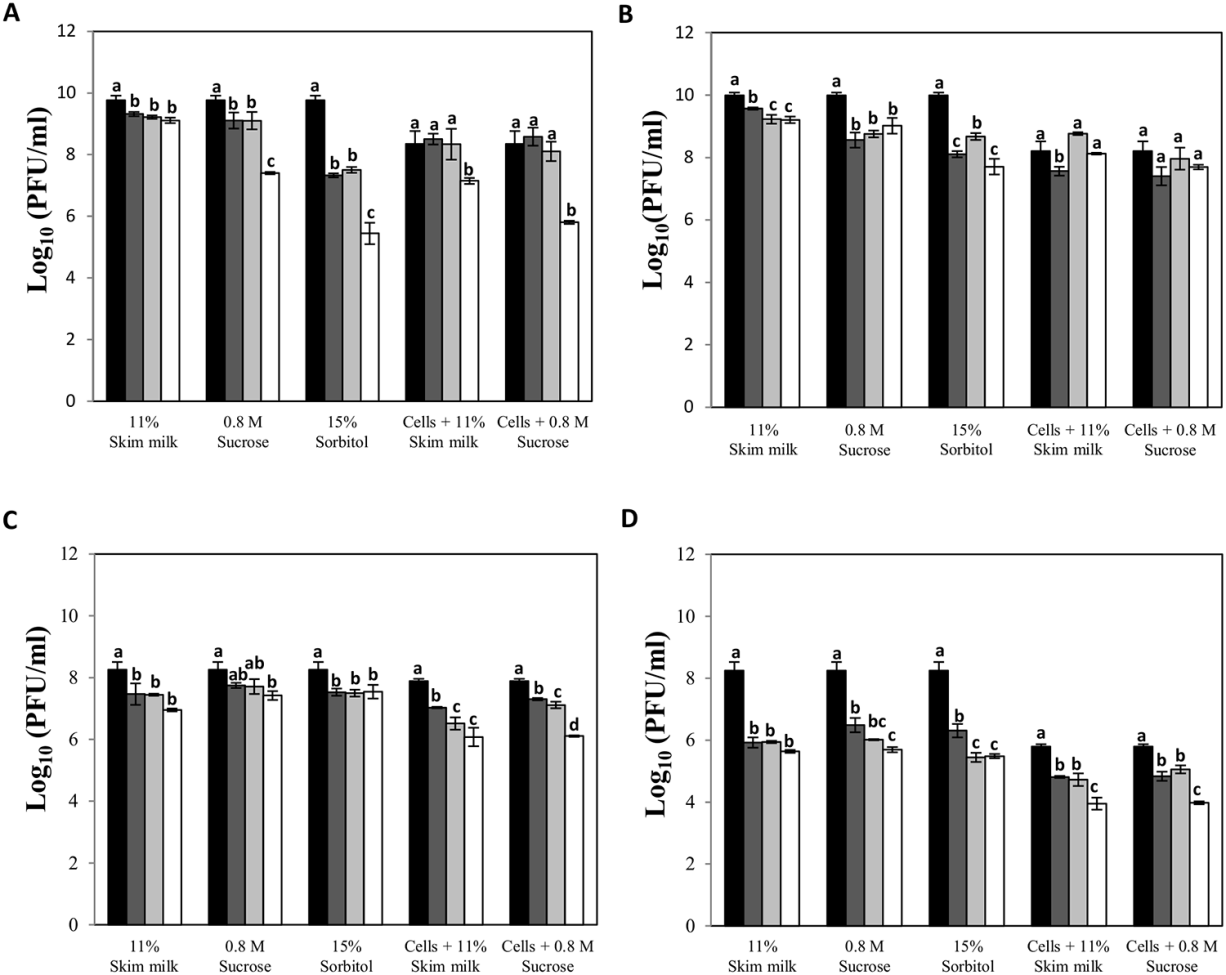
Stability of *Staphylococcus* phages during lyophilization and subsequent storage. Phages phiIPLA35 (A), phiIPLA88 (B), phiIPLA-RODI (C) and phiIPLA-C1C (D) in different additives before lyophilization (black bars), and after storage at 4°C during: 1 month (dark grey bars), 6 months (light grey bars) and 24 months (white bars). Stability of phages was expressed as the titer of samples along time. Bars represent mean ± standard deviation of phage titer obtained from three biological replicates. Different letters were significantly different in S-N-K test (P<0.01).

Looking for an alternative for long-term storage, phage lysates were freeze-dried in the presence of three stabilizers (skim milk, sucrose and sorbitol) and then stored at 4°C for 24 months. The results showed that sorbitol possessed a poor stabilizing activity for siphophages with phage titer losses of about 4.3 log units for phage phiIPLA35 (Fig 1A). However, samples corresponding to siphophages stored in skim milk retained their infectivity with a phage titer loss lower than 1 log unit. In the case of myophages, the three stabilizers conferred similar protection during lyophilization and storage (Fig 1C and 1D). Remarkably, lyophilization caused a high loss of infectivity for phage phiIPLA-C1C regardless of the stabilizer used, with reductions of 2 log units one month after the lyophilization process (Fig 1D), whereas phiIPLA-RODI showed very good stability in all cases.

As an additional method for stabilization of phages during the lyophilization process, freshly infected cells were suspended in skim milk or sucrose and further freeze-dried. Overall, protection of phages inside the infected cells proved to be an effective system to avoid the detrimental effects of the process, especially for phiIPLA-C1C since the reduction in phage titer during lyophilization was lower than those observed in other samples, i.e. the difference in phage titer between samples before and after lyophilization was 1.0 log unit, whereas using skim milk as protective the decrease reaches 2.3 log units (Fig 1D). However, higher protection was not observed along the 24 months storage period, with the exception of phiIPLA88. In samples containing infected cells, we did not observe a decrease in phage titer in comparison with the initial titer after 24 months of storage, whereas a decrease of 2.3 log units was observed for samples stored in sorbitol (Fig 1B).

### *Staphylococcus* phages retain their viability in alginate microcapsules

Stability of phages during alginate encapsulation and microencapsulation processes and subsequent storage at 4°C and 20°C was evaluated. Capsules with an average diameter size of 5 mm and microcapsules with an average diameter size of 129 μm were obtained. The phage titer calculated just after the encapsulation process was about 10^6^ PFU/ml, with the exception of capsules containing phiIPLA-C1C (Table 2).

**Table 2:** Encapsulation of *Staphylococcus* phages in alginate.

Bacteriophage	Storage conditions	Calcium alginate capsules				Calcium alginate microcapsules				SM buffer		
		After process	1 month	2 months	3 months	After process	1 month	2 months	3 months	Initial	1 month	3 months
**phiIPLA35 Log**_**10**_ **(PFU/ml)**	4°C	6.7±0.5	5.3±0.5	4.1±0.5	2.8±0.5	7.0±0.1	6.8±0.1	6.7±0.1	6.4±0.2	9.4±0.1	9.4±0.0	9.3±0.1
	20°C	6.7±0.5	-	-	-	7.0±0.1	3.4±0.1	2.0±0.7	-	9.4±0.1	9.4±0.0	8.5±0.0
**phiIPLA88 Log**_**10**_ **(PFU/ml)**	4°C	6.8±0.5	5.8±0.1	4.9±0.1	3.9±0.3	7.1±0.2	6.8±0.1	6.7±0.1	6.7±0.1	9.8±0.1	9.7±0.0	9.6±0.0
	20°C	6.8±0.5	4.1±1.1	-	-	7.1±0.2	3.9±0.1	2.4±1.2	-	9.8±0.1	9.6±0.0	8.9±0.1
**phiIPLA-RODI Log**_**10**_ **(PFU/ml)**	4°C	6.7±0.3	6.2±0.2	6.1±0.4	5.9±0.4	6.2±0.1	5.4±0.1	5.4±0.1	5.1±0.1	8.2±0.1	8.0±0.1	7.3±0.1
	20°C	6.0±0.1	5.1±0.1	3.5±0.2	-	6.2±0.1	4.2±0.3	3.1±1.1	-	8.2±0.1	7.7±0.0	6.6±0.0
**phiIPLA-C1C Log**_**10**_ **(PFU/ml)**	4°C	5.4±0.2	3.9±0.4	-	-	5.3±0.1	4.9±0.1	4.5±0.1	3.7±0.4	8.7±0.0	8.3±0.2	7.7±0.1
	20°C	5.4±0.2	-	-	-	5.3±0.1	3.4±1.2	2.7±0.2	-	8.7±0.0	7.7±0.4	7.3±0.4

Phage titer of phages (phiIPLA35, phiIPLA88, phiIPLA-RODI and phiIPLA-C1C) after alginate encapsulation and microencapsulation processes, in SM buffer and subsequent storage at 4°C and 20°C/25°C. Note: (-) below the bacteriophage detection threshold (10^2^ PFU/mL).

The storage of alginate capsules containing phiIPLA-RODI at 4°C retained the phage titer for 3 months, and this was 5.74±0.4 log units (PFU/ml) even after 6 months (data not shown). By contrast, capsules stored at 20°C maintained phiIPLA-RODI stability for 2 months with a reduction of 2.5 log units during this storage period (Table 2). The other phages were less stable at 20°C. Overall, a higher stability was observed at refrigeration temperature than at 20°C. On the other hand, microencapsulation in alginate conferred higher stability to phages compared with standard encapsulation. Thus, only about 1–1.5 units log reduction was observed after three months at refrigeration temperature. By contrast, no viable phages were detected beyond two months at 20°C (Table 2).

### PhiIPLA88 and phiIPLA-RODI can be stored as dry powder at 20°C

**Fig 2:**
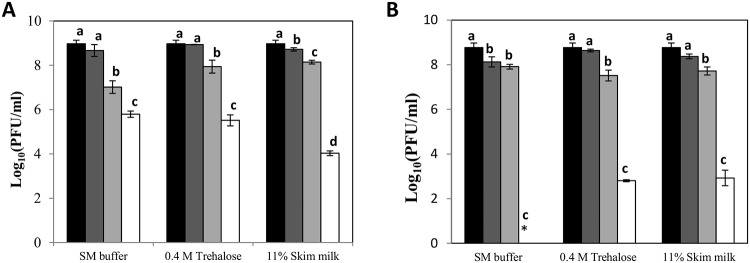
Protective effect of different stabilizers on thermal inactivation of phages. Bacteriophages phiIPLA88 (A) and phiIPLA-RODI (B), were incubated at different temperatures: 4°C (black bars), 40°C (dark grey bars), 50°C (light grey bars) and 60°C (white bars), during 30 min. Phage stability under these conditions was measured as the variation in phage titer after treatment. Bars represent mean ± standard deviation of phage titer in three biological replicates. Different letters were significantly different in S-N-K test (P<0.05). Asterisk (*): Below the bacteriophage detection threshold (10^2^ PFU/ml).

Prior to determining the effect of the spray-dryer process on phage viability, we studied the resistance of phages phiIPLA88 and phiIPLA-RODI at high temperatures in the presence of three additives (SM buffer, skim milk and trehalose). PhiIPLA88 turned out to be quite stable at 50°C and samples even maintained a titer of 10^5^ PFU/ml after 30 min at 60°C in trehalose and SM buffer (Fig 2A). Similar results were observed for phiIPLA-RODI at 40°C and 50°C, but titers were reduced by 6 log units at 60°C in the presence of trehalose and skim milk (Fig 2B).

**Fig 3:**
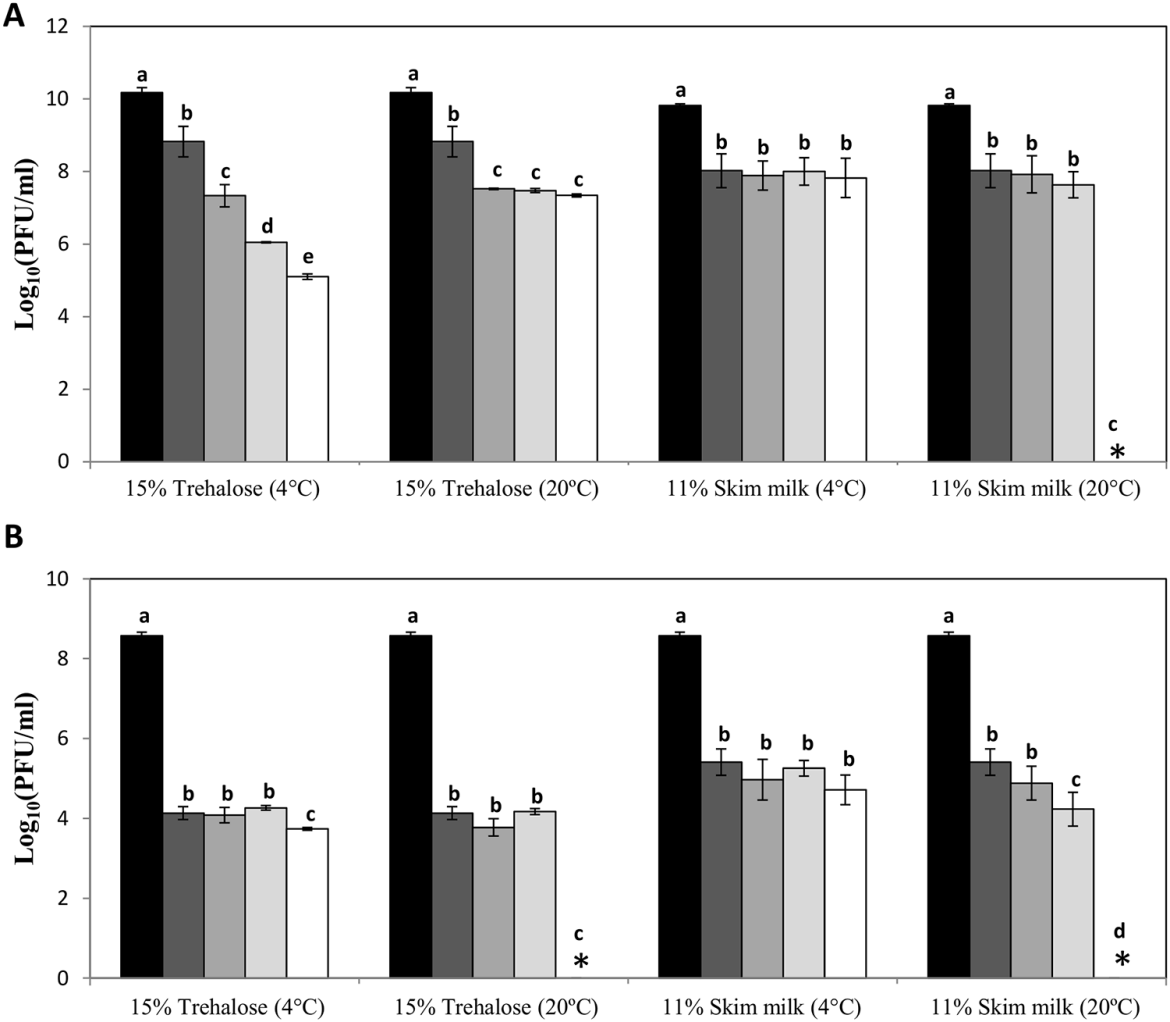
Evolution of phage titer after spray-drying process. Phages phiIPLA88 (A) and phiIPLA-RODI (B), before process (black bars), immediately after process (dark grey bars), and stored 1 month (grey bars), 6 months (light grey bars) and 12 months (white bars), at different temperatures and with different stabilizing additives. Bars represent mean ± standard deviation of phage titer in three biological replicates. Different letters were significantly different in S-N-K test (P<0.05). Asterisk (*): Below the bacteriophage detection threshold (10^2^ PFU/ml).

Based on these results, suspensions containing phiIPLA88 and phiIPLA-RODI were spray dried using skim milk and trehalose as stabilizers at 40°C as temperature for loading the samples. The resulting powders were stored at 4°C or 20°C. The samples were titrated immediately after the process and throughout 12 months (Fig 3).

A reduction of 1–2 log units was observed for phiIPLA88 in trehalose and skim milk immediately after the process (Fig 3A), whereas phiIPLA-RODI showed a greater loss of viability in both stabilizers (up to 4 log units) (Fig 3B). Regarding storage, phiIPLA88 turned out to be more stable and retained a titer of 10^7^−10^8^ PFU/ml after storage for 12 months at 20°C in samples containing trehalose as a stabilizing agent and also in samples stored at 4°C in the presence of skim milk. Unexpectedly, storage at 4°C decreased the phage phiIPLA88 titer in the presence of trehalose. Finally, despite the lower stability during the process, phiIPLA-RODI also maintained infectivity at 20°C for six months and at refrigeration temperature for 12 months regardless of the stabilizer (Fig 3B).

## Discussion

Commercialization of bacteriophage-based products is expected to increase over the next few years due to the implementation of phage therapy to overcome the global antibiotic resistance crisis [33]. In fact, several clinical trials using bacteriophages to treat infectious diseases in humans have been undertaken [34–37], and it is also possible that food industries will incorporate these products in their routine disinfection procedures [38]. In this context, it is still necessary to optimize phage storage conditions for both small stocks intended preserve phages over prolonged periods of time and, large volumes of purified phages ready for delivery. Accordingly, this study compares the stability of small stocks of four *Staphylococcus* phages over a 24-month period using three preservation techniques (low temperature, lyophilization and encapsulation).

Low-temperature preservation techniques have been widely used to maintain phage stocks during long-term periods [39]. They have the advantage of being affordable for most laboratories although the main drawback is their associated energy cost and their unsuitability for large volumes. Here, we have shown a clear relationship between storage temperature and phage stability, which was particularly evident for phages belonging to the *Myoviridae* family. Thus, phage stability increased as storage temperature decreased. In fact, we observed that the reduction in phage titer was less than 1.5 log units after 12 months at -196°C, regardless of the phage and the stabilizer used. Obviously, this technique is only useful for preservation of small volumes of master stock. The results obtained at -80°C were similar, suggesting that phages from both families can be preserved at this temperature in any stabilizer for long-term storage (at least 2 years).

Some differences in stability between the two phage families were detected at -20°C. Previous studies indicate that the phage titer reductions observed in frozen samples stored at -20°C is mainly related with the formation of large ice crystals [9]. Our results suggest that this effect was more pronounced for phages belonging to the *Myoviridae* family despite the presence of stabilizers. More specifically, phiIPLA-RODI and phiIPLA-C1C were relatively unstable in the presence of trehalose and sucrose, disaccharides that are commonly used for the cryopreservation of microorganisms [40]. By contrast, it is worth noticing the protective effect of skim milk and glycerol at this temperature. Indeed, glycerol showed a good efficacy for phiIPLA-RODI preservation. One possible explanation is that the cryopreservation effect of disaccharides is lost during the slow freezing [41].

Regarding the preparation of master stocks to be used for industrial phage productions, in general, storage in infected cells did not provide enhanced protection compared with naked phages. Overall, our results suggest a lesser effectiveness of this method for our staphylococcal phages than was previously observed for *S*. *aureus* phages A5W and phiAGO1.3, in which no titer reduction was detected [31]. These differences could be attributed to the longer storage time and the higher temperature (-20°C) at which our phages were subjected compared with A5W and phiAGO1.3. In addition, the differences in the specific conditions used for obtaining infected cells (MOI, time for adsorption, washing steps after infection) cannot be ruled out as an additional cause of the lower phage viability observed in our phages.

For long-term preservation of *Staphylococcus* phages we also explored lyophilization, which has been proven efficient for other bacteriophages [42, 10, 11, 43, 44]. Although specific equipment is required for sample preparation, this method offers the advantage of producing a dry powder that can be easily stored and shipped. After lyophilization, samples containing phages were stored at 4°C to avoid the instability previously observed for some bacteriophages in powders containing trehalose due to matrix crystallization [45]. Skim milk provided good protection along 24 months for all the phages studied, although a notable reduction in phage titer occurred for phiIPLA-C1C during the lyophilization process. Studies about stability of other *Myoviridae* phages such as the *S*. *aureus* phage ISP also confirmed the sensitivity of these phages to the lyophilization process and showed disaccharides trehalose and sucrose as effective stabilizers [44]. In this context, Puapermpoonsiri et al. [42] found that the stability of lyophilized bacteriophages is clearly dependent of the moisture content of the powder, the optimal being in a range of 4–6%.

Microencapsulation of bacteriophages is a technology mainly explored to protect phages from the acidic environment found in the gastrointestinal tract when these phages are used in treatment of human or animal infections [46, 47]. Here, we evaluated the use of alginate encapsulated phages to improve their stability under storage and shipping conditions. Our results showed that microencapsulated phages could be maintained at 20°C for 2 months, which facilitates transportation of the samples. For long-term storage, however, microencapsulation did not result in improved phage viability compared with the viability obtained with low temperature (including refrigeration) and lyophilization methods. Of note, microencapsulation techniques have an extra cost that should be evaluated depending on final application. In spite of this, their higher resistance to environmental conditions might compensate for these drawbacks. A variant of the microencapsulation technique, using microfluidic devices, has been recently used to produce calcium alginate capsules containing the bacteriophage UFV-AREG1, which is applied in the sanitization of food surfaces [14]. Similarly, this technique was used for encapsulation of the *Clostridium difficile* bacteriophage CDKM9 intended for treatment of colon diseases [48].

Looking for a less harmful strategy to maintain phage viability, and considering its feasibility to be adapted to industrial scale with continuous production, we evaluated the spray drying technique. The need to use high temperatures to dry phage suspensions implies the imperative use of protective compounds to avoid denaturing of phage proteins. For this purpose, both trehalose and skim milk were selected as they have shown the capacity to protect proteins from denaturation [49, 50]. Our results showed a higher stability of phiIPLA88 compared to that of phiIPLA-RODI in both stabilizers. Moreover, reductions in phage titer observed after the drying process were similar to those described for the *Staphylococcus* myophage Romulus subjected to a similar process using trehalose [51]. Definitely, further optimization of the spray-drying process is necessary to reduce the decline in viability caused by the hot air used to dry the phage suspension. For instance, the use of a low-temperature spray-drying system allows obtaining an inhalable powder containing phages to treat pulmonary infections [52, 53]. In this context, it has already been reported that bacteriophages belonging to different families behave differently when subjected to aerosolization [54]. Future work is required to explore other stabilizers, whose characteristics would not only allow long-term storage of phage suspensions but also their direct delivery in applications such as phage therapy or biocontrol.
